# Multicenter Evaluation of Processing and Analysis of College of American Pathologists (CAP) Proficiency Testing Samples by Laboratory Automation

**DOI:** 10.1128/JCM.03233-20

**Published:** 2021-04-20

**Authors:** N. Esther Babady, Lori Bourassa, Carey-Ann D. Burnham, Mark Fisher, Erin McElvania, Christopher R. Polage, Julie Ribes

**Affiliations:** a Department of Laboratory Medicine, Memorial Sloan Kettering Cancer Center, New York, New York, USA; b Department of Laboratory Medicine & Pathology, University of Washington, Seattle, Washington, USA; c Department of Pathology & Immunology, Washington University in St. Louis School of Medicine, St Louis, Missouri, USA; d Department of Pathology, University of Utah School of Medicine, ARUP, Salt Lake City, Utah, USA; e Department of Pathology, NorthShore University Health System, Evanston, Illinois, USA; f Department of Pathology, Duke University Health System, Durham, North Carolina, USA; g Department of Pathology and Laboratory Medicine, University of Kentucky, Kentucky, USA; h Department of Medicine, Memorial Sloan Kettering Cancer Center, New York, New York, USA; Medical College of Wisconsin

**Keywords:** CLIA, automation, bacteriology, proficiency testing

## LETTER

To maintain their Clinical Laboratory Improvement Amendment (CLIA) accreditation, clinical laboratories are expected to enroll in proficiency testing (PT) to confirm that all parts of the diagnostic testing process perform as expected (1). In recent years, an increasing number of clinical microbiology laboratories have adopted laboratory automation for processing and incubation of specimens submitted for bacterial culture (2). Some laboratories may not be processing PT samples on their automated systems and, per CLIA regulations, “PT specimens must be tested with the laboratory’s regular workload, using routine methods, and testing the PT specimens the same number of times it routinely tests patient specimens” (1). Given that PT samples are expected to be tested in the same manner as clinical samples, we sought to examine the performance of the two current laboratory automation systems, the Copan WASPLab (Copan Diagnostics, Murrieta, CA) and the Beckton Dickson Kiestra Total Lab Automation (TLA) (BD and Company, Franklin Lakes, NJ), for processing and incubation of PT samples from the College of Pathologists (CAP) bacteriology PT survey for 2020.

This was a multicenter, retrospective study conducted at seven sites across the United States, including three laboratories with BD Kiestra TLA and four laboratories with WASPLab. Swabs from the first two CAP D surveys for 2020 (the A and B surveys), which occurred between February and June 2020, were used for the study. All laboratories performed both manual and automated processing. The extra swab was used for the study only after submission of the PT results. Each CAP PT swab was added directly to an ESwab transport tube containing 1 ml of Amies medium (Copan Diagnostics, Murrieta, CA) and vigorously mixed manually. The swab was rubbed against the side of the ESwab vial and removed, and the vial processed per standard protocols in each laboratory. This process differed from the CAP PT instructions, in which the PT swab is submerged in a rehydration fluid for several seconds with mixing to wet the swab prior to manual inoculation of the medium.

Culture plates were incubated, imaged, and interpreted using the automated system following each laboratory's individual protocol. Incubation time and imaging time points varied by laboratory and culture type, with incubation lasting up to 120 h and the first image taken as early as 10 h after inoculation. Preliminary or final identifications were recorded, and bacterial growth quantity provided as 1+ (<10 colonies/plate or 1st quadrant growth only), 2+ (10 to 50 colonies/plate or growth in the 1st and 2nd quadrant only), 3+ (50 to 300 colonies/plate or growth in 1st to 3rd quadrant), or 4+ (>300 colonies/plate or growth in all quadrants).

**FIG 1:**
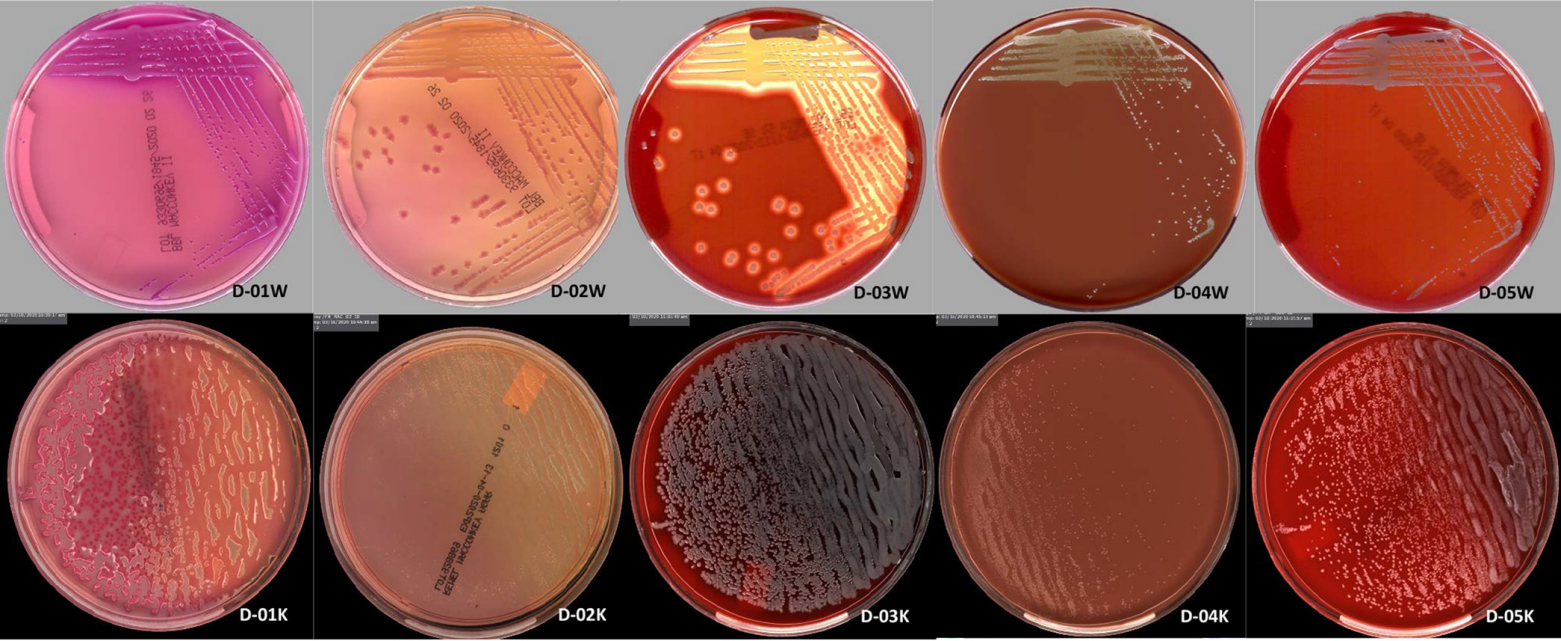
Representative images of PT samples from WASPLab and Kiestra total lab automation (TLA). Images shown were captured at either 18 h (Kiestra TLA) or 24 h (WASPLab). Survey D-A images (D-01 to D-05) with D-01W-D-05W images captured on the WASPLab (24 h) and D-01K-D-05K images captured on the Kiestra TLA (18 h).

Laboratories 1, 2, and 7 performed both surveys D-A and D-B, laboratories 3, 4, and 5 performed the D-A survey only, and laboratory 6 performed survey D-B only. There was complete agreement in the type of organism recovered (e.g., Gram-negative bacilli) for all the laboratories and between manual and automated processes in all laboratories (data not shown). Agreement was also seen between both automated platforms and with the expected CAP PT results, suggesting that processing and incubation using the automated systems did not negatively impact the PT samples (Table 1). The quantities of organisms recovered were within ± 1 category for all but two isolates: *Streptococcus* was present in quantities of 4+ in two laboratories, 3+ in one laboratory, and 2+ in one laboratory, while coagulase-negative *Staphylococcus* was present in 1+ quantity in one laboratory and 4+ in all other laboratories. These differences could be due to variation in isolate stability over the course of storage prior to the study at the corresponding laboratory, or differences in quantity of organisms present in the amount of sample inoculated. Of note, only the bacterial identification of the PT is graded. Possible contaminants were recovered (but not reported) in one laboratory but not in the others. The same contaminants were recovered when the PT samples were plated manually. Representative images (survey D-A) from the different automation systems show similar results for images taken after either 18 h or 24 h of incubation (Fig. 1).

**TABLE 1:** CAP proficiency testing survey D-A (numbers 1 to 5) and D-B results (numbers 8 to 12)^*a*^

PT no.	1-WASPLab	2-WASPLab	3-WASPLab	4-WASPLab	5-Kiestra TLA	6-Kiestra TLA	7-Kiestra TLA
1	4+ K. pneumoniae	4+ K. pneumoniae	4+ K. pneumoniae	4+ K. pneumoniae	4+ K. pneumoniae	TNP	4+ K. pneumoniae
2	4+ P. aeruginosa; 4+ CoNS	4+ P. aeruginosa; 4+ CoNS	3+ P. aeruginosa; 4+ CoNS	4+ P. aeruginosa; 1+ CoNS	4+ P. aeruginosa; 4+ CoNS	TNP	4+ P. aeruginosa; 4+ CoNS
3	4+ S. aureus	4+ S. aureus; 2+ Presumptive diphtheroids*	4+ S. aureus	4+ *Staphylococcus*	4+ S. aureus	TNP	4+ S. aureus
4	4+ *Streptococcus*	3+ *Streptococcus*	2+ *Streptococcus*	4+ Beta hemolytic *Streptococcus*	4+ *Streptococcus*	TNP	4+ *Streptococcus*
5	4+ CoNS	4+ CoNS	4+ CoNS	4+ CoNS	4+ CoNS	TNP	4+ CoNS
8	4+ *Cronobacter*	4+ *Cronobacter*	TNP	TNP	TNP	4+ *Cronobacter*	4+ *Cronobacter*
9	4+ GBS; 1+ *C. septicum*	4+ GBS; 1+ *C. septicum*	TNP	TNP	TNP	TNP	4+ GBS; 2+ *C. septicum*
10	4+ A. haemolyticum	4+ A. haemolyticum; 2+ *Neisseria* sp.^*b*^	TNP	TNP	TNP	4+ A. haemolyticum	4+ A. haemolyticum
11	3+ P. multocida; 1+CoNS	4+ P. multocida; 2+ CoNS	TNP	TNP	TNP	4+ P. multocida; 1+ CoNS	4+ P. multocida; 1+ CoNS
12	>100,000 E. coli	>100,000 E. coli	TNP	TNP	TNP	>100,000 E. coli	>100,000 E. coli

a CoNS, coagulase negative staphylococci; GBS, group B *Streptococcus*; TNP, testing not performed; TLA, total lab automation.

b Additional organisms recovered but not reported.

While the sample size was relatively small and a limitation of the study, the data from this multicenter study show that CAP bacteriology PT samples are compatible with both of these laboratory automation systems and results were in line with both the manual set-up and expected CAP PT results. Processing and analysis of proficiency testing samples can and should be performed as done by the laboratory for patient specimens, including automated processing and reading if using either of these systems. Regulatory agencies such as CAP might consider including processing instructions compatible with laboratory automation as part of the kit instructions.

## References

[1] Code of Federal Regulations. 2020. Title 42. Public health. Chapter IV. Centers for Medicare & Medicaid Services, Department of Health and Human Services. Subchapter G. Standards and certification. Part 493. Laboratory requirements. Subpart I. Proficiency testing programs for nonwaived testing. 42 CFR 493.9.

[2] Bailey AL, Ledeboer N, Burnham CD. 2019. Clinical microbiology is growing up: the total laboratory automation revolution. Clin Chem 65:634–643. 10.1373/clinchem.2017.274522. 30518664

